# Low avidity of human papillomavirus (HPV) type 16 antibodies is associated with increased risk of low-risk but not high-risk HPV type prevalence

**DOI:** 10.1186/1756-0500-4-170

**Published:** 2011-06-06

**Authors:** Proscovia B Namujju, Lea Hedman, Klaus Hedman, Cecily Banura, Edward K Mbidde, Dennison Kizito, Romano N Byaruhanga, Moses Muwanga, Reinhard Kirnbauer, Heljä-Marja Surcel, Matti Lehtinen

**Affiliations:** 1 National Institute for Health and Welfare, Oulu, Finland; 2 Uganda Virus Research Institute, Entebbe, Uganda; 3 School of Public Health, University of Tampere, Finland; 4 Department of Virology, University of Helsinki and Huslab, Helsinki, Finland; 5 College of Health Sciences, Makerere University, Kampala, Uganda; 6 Department of Obstetrics and Gynecology, San Raphael of St. Francis Hospital, Nsambya, Uganda; 7 Department of Public Health Sciences, Karolinska Institute, Stockholm, Sweden; 8 Entebbe Hospital, Entebbe, Uganda; 9 Department of Dermatology, Medical University Vienna, Austria

**Keywords:** antibody, avidity, genital infection, HPV, prevalence

## Abstract

**Background:**

Low avidity of antibodies against viral, bacterial and parasitic agents has been used for differential diagnosis of acute versus recent/past infections. The low-avidity antibodies may however, persist for a longer period in some individuals.

**Findings:**

We studied the association of human papillomavirus (HPV) type 16 antibody avidity with seroprevalence to HPV types 6/11/18/31/33/45. Antibody avidity was analysed for 365 HPV16 seropositive pregnant Finnish and Ugandan women using a modified ELISA.

Low avidity of HPV16 antibodies was found in 15% of Finnish and 26% of Ugandan women. Ugandan women with low-avidity HPV16 antibodies had an increased risk estimate for HPV6/11 (odds ratio, OR 2.9; 95%CI 1.01-8.4) seropositivity but not to high-risk HPV types 18/31/33/45.

**Conclusion:**

Association of the low avidity HPV16 antibody "phenotype" with possible susceptibility to infections with other HPV types warrants investigation.

## Background

Persistent infections with high-risk human papillomavirus (hrHPV) type 16 cause half of cervical cancer (CxCa) morbidity/mortality [1,2]. Infections with multiple hrHPVs further increase the CxCa risk, and promote progression of cervical intraepithelial neoplasia (CIN) [3,4]. Vaccines against HPV16 and HPV18 (HPV16/18) have high protective efficacy against infections with the vaccine and some non-vaccine HPV types (31/45) and associated CIN [5-7]. Various other non-vaccine HPV types 33/35/52/58 are, also however, relatively prevalent in Finland and Uganda [8-11].

HPV16 causes genital infections. Following the infection, development of antibody response takes from 6 to 18 months [12,13]. During this time, HPV is resolved through T helper cell activation of cytotoxic T cells and B cells to produce neutralizing IgG antibodies [12]. Immune response eliminates HPV in 90% of infected women [4].

Maturation of the IgG antibody avidity takes approximately 6 months [14-16]. This is used in the distinction of acute and recent/past infections with, e.g., toxoplasmosis, rubella, and parvovirus [17-20]. On the other hand, low-avidity antibodies have been found (outside the 6 months period) in chronic infections, e.g., cytomegalovirus (CMV) and HPV16 [21-23].

We evaluated whether presence of low-avidity of HPV16 antibodies is associated with an increased risk of prevalence for other HPV types.

## Material and methods

### Participants

A total of 4748 pregnant Finnish (2784) and Ugandan (1964) women participated in an epidemiological study [10]. In Finland, all pregnant women donate serum samples to Finnish Maternity Cohort (FMC) for the screening of congenital infections and consent to further serological use of the samples for health-related research [10,24]. In Uganda all participating women consented for the use of the samples to serological HPV and other sexually transmitted infections research [10]. The study was approved by the institutional review boards at the National Institute for Health and Welfare (THL), Finland; Uganda Virus Research Institute; St Raphael of St Francis Hospital Nsambya, and Uganda National Council of Science and Technology.

### Laboratory analysis

*Chlamydia trachomatis *and HIV antibodies, and serum cotinine (current smoking > 20 ng/ml) were analysed by ELISA as described [10]. Standard ELISA for HPV6/11/16/18/31/33/45 antibodies was used [10,24-27], with modifications [17-19] for HPV16 antibody avidity analysis. Briefly, Nunc™ micro plates were coated with HPV16 VLPs (kindly provided by Kathrin Jansen, Merck Research Labs, Philadelphia, PA) by overnight incubation at 4°C. Samples were serially diluted: 1:1, 1:4, 1:16, 1:64, 1:256 in phosphate-buffered saline (PBS) with 10% fetal bovine serum (blocking buffer, BB). After blocking the plate, 50 ul of diluted samples were added to wells A-D (1:4,1:16,1:64,1:256) and wells E-H (1:1,1:4,1:16,1:64). To columns 1, 2 and 3, blank, low and high avidity controls were added and incubated overnight at 4°C [28]. Wells A-D were washed 3 times with 200 ul of PBS/0.05% Tween 20 (PBS + T), and wells E-H were washed with 6M urea (Promega, Biofellows, Finland) in PBS. Each wash was for 5 min. All wells were again washed two times with PBS + T, and wells E-H, treated with the 6M urea, were washed three extra times to remove excess urea. The primary and secondary antibodies were incubated for 90 and 60 min. An ABTS-substrate was added and the reaction stopped after 40 min. Optical densities (OD) of each control and individual samples were plotted: Curve 1: OD from urea wash and Curve 2: OD from PBS + T wash. Distance between the curves at OD 0.2 (IgG threshold) was measured and matched with corresponding avidity index [17-20]. Cut-off for low-avidity (30%) was calculated from mean + 3 SD of low-avidity controls added on each of the 40 plates, according to standard procedures [18-20]. The low avidity controls were identified by repeated measurements from sera obtained one month post third dose in conjunction of an HPV vaccination study.

### Statistical analysis

Logistic regression was used to calculate the relative risk (odds ratio, OR, with 95% confidence intervals, 95%CI) of being HPV6/11/18/31/33/45 seropositive associated with low avidity HPV16 antibodies. Single HPV16 seropositives were the reference group. Adjustment was stepwise for age, *Chlamydia trachomatis *and HIV antibodies, and cotinine. Statistical analyses were done using Stata 8 (College Station, TX).

## Results and Discussion

Overall, HPV16 seroprevelances were equal (21%) in both countries [10]. Altogether 365 of the 994 originally HPV16 seropositive women [10] were randomly selected. Majority (248) were Finns (mean age of 22 years), 117 were Ugandans (mean age 23 years). Among the Finns 113 had HPV16 antibodies only, 36 had HPV16+ HPV6 or HPV11 antibodies, 59 had HPV16+ HPV31 or HPV33 antibodies, and 40 had HPV16+ HPV18 or HPV45 antibodies. Among the Ugandans 45 had HPV16 antibodies only, 32 had HPV16+ HPV6 or HPV11 antibodies, 24 had HPV16+ HPV31 or HPV33 antibodies, and 16 had HPV16+ HPV18 or HPV45 antibodies (Table 1).

**Table 1:** Association (odds ratio, OR, with 95% confidence interval, CI) of low avidity HPV16 antibodies (ab) with increased risk of being seropositive for other HPV types among Finnish and Ugandan women.

Population	Seropositivity	Total	Prevalence of low ab-avidity (%)	Crude OR (95% CI)
Finland (n = 248)	HPV16 only	113	19 (7.7%)	1
	HPV16 + 6 or 11	36	8 (6.3%)	1.4 (0.6-3.6)
	HPV16 + 31 or 33	59	5 (2.5%)	0.5 (0.2-1.3)
	HPV16 + 18 or 45	40	6 (2.2%)	0.9 (0.3-2.4)
Uganda (n = 117)	HPV16 only	45	9 (7.7%)	1
	HPV16 + 6 or 11	32	15 (12.8%)	3.5 (1.3-9.7)
	HPV16 + 31 or 33	24	4 (3.4%)	0.8 (0.2-2.9)
	HPV16 + 18 or 45	16	2 (1.7%)	0.6 (0.1-4.0)
All (n = 365)	HPV16 only	158	28 (7.7%)	1
	HPV16 + 6 or 11	68	23 (6.3%)	2.4 (1.3-4.6)
	HPV16 + 31 or 33	83	9 (2.5%)	0.6 (0.3-1.3)
	HPV16 + 18 or 45	56	8 (2.2%)	0.8 (0.3-1.8)

All = combined data of Finnish and Ugandan women

Cutoff_30% = using low avidity controls mean plus 3 standard deviations

**Figure 1:**
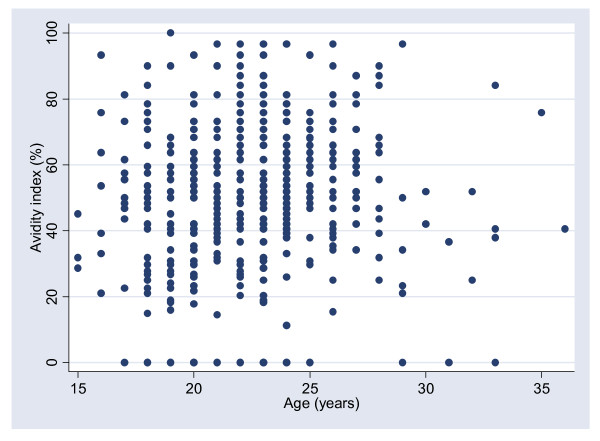
**Correlation between antibody avidity and age (Pearson correlation co-efficient 0.07, p = 0.15)**.

Overall, the prevalence of low-avidity HPV16 antibodies was 15% (38/248) among the Finns and 26% (30/117) among the Ugandans (p < 0.0001). There was no statistically significant correlation (Pearson correlation co-efficient 0.07, p = 0.15) between HPV16 antibody avidity and age (Figure 1). The seroprevalences of HIV, *C. trachomatis*, and smoking were 0%, 25% and 23% (16/69, cotinine data available) among the Finns and 9%, 27% and 2% (2/111, questionnaire data available) among the Ugandans.

HPV6/11 seropositivity was increased in women who had low-avidity HPV16 antibodies (crude OR 2.4, 95%CI 1.3-4.6). After stratification by country and adjustment, increased risk for being HPV6/11 seropositive was found only among the Ugandans (adjusted OR 2.9, 95% CI 1.01-8.4). Estimates on the association of low-avidity with seropositivity for other hrHPVs were not different from unity (Table 2).

**Table 2:** Association (adjusted odds ratio, OR, with 95% confidence interval, CI) of low avidity HPV16 antibodies with increased risk of being seropositive for other HPV types among Finnish and Ugandan women.

Population	Seropositivity	**OR**^**1 **^**(95% CI)**	**OR**^**2 **^**(95% CI)**	**OR**^**3 **^**(95% CI)**
Finland (n = 248)	HPV16 only	1	1	1
	HPV16 + 6 or 11	1.1 (0.4-2.8)	1.9 (0.6-6.1)	0.8 (0.1-6.8)
	HPV + 31 or 33	0.3 (0.1-1.0)	0.3 (0.1-1.3)	0.8 (0.0-1.0)
	HPV + 18 or 45	0.7 (0.2-1.8)	0.5 (0.1-2.0)	0.3 (0.2-3.8)
Uganda (n = 117)	HPV16 only	1	1	1
	HPV16 + 6 or 11	3.5 (1.3-9.7)	3.0 (1.1-8.5)	2.9 (1.0*-8.4)
	HPV + 31 or 33	0.8 (0.2-2.9)	0.7 (0.2-2.5)	0.6 (0.2-2.3)
	HPV + 18 or 45	0.6 (0.1-3.0)	0.5 (0.1-2.9)	0.5 (1.0-2.8)

Cutoff_30% = using low avidity controls mean plus 3 standard deviations

OR^1 ^= adjusted for age

OR^2 ^= adjusted for age, *Chlamydia trachomatis, HIV*

OR^3 ^= adjusted for age, *C. trachomatis, HIV *and smoking

*1.0 = lower 95% confidence limit 1.01

HPV16 seropositivity is associated with increased risk of acquiring other HPVs [10,26,29]. The prevalence of low-avidity of HPV16 antibodies was 15% and 26% among pregnant Finnish and Ugandan women, respectively. Sexual risk-taking behavior also predisposes women to various HPV types earlier in Uganda than in Finland [10]. Thus, the Ugandans had more time to get infected with both HPV16 and other HPV infections than the Finns. There were, however, higher proportions of HPV16 seropositive Ugandans with low-avidity HPV16 antibodies and increased risk of HPV6 or 11 prevalence.

Low-avidity antibodies induced by microbial antigens alone are less variable and less functionally versatile than those induced with T-cell help [14,30]. In immuno-compromized patients low-avidity cytomegalovirus antibodies can persist for several months [21,22]. Comparable delayed maturation of HPV16 antibody avidity, probably associated with impaired ability to produce high quality neutralizing antibodies, identified women with increased risk for HPV6 or HPV11 seropositivity, possibly due to increased susceptibility. On the other hand, the first evidence from the HPV vaccination studies showed that following HPV vaccination, there was no correlation between avidity and neutralization capacity of the vaccine induced antibodies [31]. Why only the lrHPV occurrence was increased in our study remained unclear.

HPV16 seropositives only were eligible for the study. Thus, due to moderate sensitivity (75%) of HPV16 serology [28] some HPV16 infected women could have been excluded. Moreover, even if HPV seropositivity is an indicator of cumulative incidence of HPV infections [12,25,28] our cross-sectional approach did not reveal the order of acquiring the various HPV infections.

The association was seen only in the Ugandans, whose HIV seroprevalence was 9%. Adjusting for HIV, however, had no effect on the low-avidity associated risk, antibody avidity is not affected by HIV infection [21,22]. Smoking, which impairs HPV antibody response [27], might, however, intervene with the development (maturation) of antibody avidity as the observed association almost lost statistical significance after adjusting for smoking. In conclusion, association of the low avidity HPV16 antibody "phenotype" with possible susceptibility to other HPV infections warrants investigation.

## Conflict of interests

PBN, CB, LH, KH, EKM, RB, MM, RK, HMS declare no conflicts of interest. ML has obtained grants from Merck&Co. Inc. and GSK-Biologicals for HPV vaccination studies through his employers.

## Authors' contributions

PBN, designed, ran the study, conducted data collection and analysis and wrote the report.

ML designed the study, supervised PBN in data analysis, data interpretation and report writing. KH, developed the modified ELISA. KH, LH, HMS supervised PBN in laboratory analysis and contributed to data interpretation and report writing. KD processed the samples. EKM, CB, RB, MM, RK, contributed to study design. All authors read and approved the final manuscript.
